# Defining Telehealth for Research, Implementation, and Equity

**DOI:** 10.2196/35037

**Published:** 2022-04-13

**Authors:** Joy Roy, Deborah R Levy, Yalini Senathirajah

**Affiliations:** 1 Department of Biomedical Informatics School of Medicine University of Pittsburgh Pittsburgh, PA United States; 2 Department of Medical Informatics and Clinical Epidemiology Oregon Health Sciences University Portland, OR United States

**Keywords:** telehealth, telemedicine, standards, health equity, public health, digital health, delivery of health care

## Abstract

When the COVID-19 pandemic spurred a disruption in health care delivery, the role of telehealth shifted from an option to a near necessity to maintain access when in-person care was deemed too risky. Each state and many organizations developed temporary telehealth policies for the COVID-19 emergency, each policy with its own definitions, coverage, government cases, and regulations. As pandemic-era policies are now being replaced with more permanent guidelines, we are presented with an opportunity to reevaluate how telehealth is integrated into routine health care delivery. We believe that the timing and nature of the sequential steps for redefining telehealth are critical and that it is important to develop a clear and agreed-on definition of telehealth and its components at this time. We further suggest a necessary preliminary step is to support clear communication and interoperability throughout the development of this definition. Precise and standardized definitions could create an unambiguous environment for clinical care for both patients and providers while enabling researchers to have more precise control over their investigations of telehealth. A consensus when defining telehealth and its derivatives at this critical stage could create a consistent expectation of care for all patients and those who set the standards of care, as it has for other clinical scenarios with clear guidelines.

## Introduction

The pandemic accelerated the swift adoption of telehealth [1-4]. As the emergency mode of health care delivery during the pandemic draws to a close for many entities and organizations, questions regarding sustainability, definitions of equitable access, and the future of telehealth arise [3]. Between June 6 and November 6, 2020, nearly one-third of all health visits were conducted remotely as telehealth visits, a considerable expansion compared to prior years [1]. Although 43% of hospitals reported the capacity of supporting telemedicine in 2019, 95% of health centers reported using telehealth during the COVID-19 pandemic, which reflected a rapid increase in use [1]. However, the emergency adoption of telehealth has led to widely differing practices, varying definitions of what constitutes telehealth or telemedicine, and a spectrum of state rules; this variability may be linked to the rapid adoption of telemedicine during the pandemic emergency phase. This new and expanded adoption has at times included exemptions from the usual regulatory processes; these exemptions are now being reevaluated [5,6]. The end of the emergency adoption period opens an opportunity to reevaluate the standards and definitions of telehealth and its components and may reveal the more subtle risk of perpetuating suboptimal practices adopted during the emergency phase of the pandemic.

We will employ the World Health Organization (WHO) definition of equity which explains that equity “is the absence of unfair, avoidable, or remediable differences among groups of people, whether those groups are defined socially, economically, demographically, or geographically or by other dimensions of inequality (e.g., sex, gender, ethnicity, disability, or sexual orientation)” [7]. We propose that a clear and standardized definition of telehealth, ideally developed through consensus across a wide variety of organizations, must be established to support better implementation and evaluation and to contribute to greater equity in health care access overall.

## Definition

To define telemedicine, some sources distinguish between telehealth and telemedicine, while other sources use the terms interchangeably [8-14]. Telemedicine definitions commonly agree that it encompasses the remote diagnosis and treatment of patients by telecommunications infrastructure, while telehealth is commonly considered a superset of telemedicine, defined as any services used to provide care remotely (eg, live conferencing, remote patient monitoring, and personal health apps) [8,9,11]. Already, the terms telemedicine and telehealth both appear to leave room for interpretation and have a broad scope; telemedicine is often the narrower of the 2 terms, though still vaguely defined. With the introduction of derivative terms such as mHealth and eHealth [15], coupled with the different technologies (eg, video visit, audio-only visit, and email- and text-based correspondence) and communication methods (synchronous or asynchronous) that may be used for a telemedicine visit, the expectations for what types of care telemedicine encompasses are further obfuscated.

Standardized definitions in clinical disciplines, as in other disciplines, can help categorize information and encourage more precise communication relating to the area of interest [16-18]. We argue that this situation presents an opportune time to establish a standardized definition of telehealth, with explicit classifications of its modalities, applications, scope, and relationships to other modes of health care. Prior to the expansion of telehealth during the pandemic, telehealth had limited applications in both urban and rural settings, including but not limited to tele–critical care and tele-neurology applications [4,19,20]. Patients communicating with providers and having clear expectations when doing so is known to improve engagement in care; this could be an indirect impact of improving definitions in the field of telehealth [21,22]. As telehealth expands, knowing what the term constitutes will enable more precise communication between providers, patients, policy makers, and researchers.

Clear definitions could support patients in scheduling and requesting visits with greater clarity of the scope of telehealth and expectations of what will occur during their visit as well as potential outcomes to anticipate at the end of the visit. Providers can also benefit by recommending services that stand to offer the best anticipated utility given the combination of patients’ conditions, needs, and expectations. For example, video visits are not always possible or even desired by patients [23]. However, phone calls or chat box correspondence may not be enough for clinicians to gauge a patient’s health properly for a specific condition. Another factor can be what is covered by a payor or state’s coverage for telehealth-related services. Hence, with greater control of the nomenclature used to describe telehealth services, providers can work with patients to recommend the most appropriate type of care, which can lead to greater outreach to patients, quality of care, and return on investments for both patients and physicians. Westby et al [24] provide a case of a patient who had a telehealth visit that can offer an example of the importance of matching expectations with patient understanding of what is offered. Because the patient had concerns regarding his ability to use the technology for a video visit, the clinic staff scheduled the patient for a telephone visit. This resulted in several potentially unanticipated benefits to both the patient and provider, including the patient being comfortable disclosing his challenges with reading, which the providers were unaware of, and the patient being able to spell his medication names directly from the bottles to the provider. The providers were able to clarify the instructions for his medications, which the patient misread due to his difficulty with reading. Audio-only visits are generally not considered telehealth visits in every state or institution, whereas video visits are accepted as telehealth universally [11]. Westby et al [24] note that the Center for Medicare and Medicaid, at the time, had a narrower definition of telehealth than the WHO and excluded telephone visits from reimbursement. Had the clinic’s staff not been precise about describing the nature of the visit at the time of scheduling with the patient, and instead simply scheduled a “telehealth visit,” the patient could have assumed it to be a video visit and may have chosen to cancel the appointment without the barriers to his participation being brought to light. Patients may not be aware of the options and accessibilities that are offered to them, especially when every state, institution, and payor may have different stipulations of what constitutes a telehealth visit.

Similar arguments regarding the need for a standardized interpretation of telehealth have been made previously, with attempts to develop a common “taxonomy of telemedicine,” but this has not gained widespread adoption [18]. Regulation and credentialing are governed by state governments, where each state defines telehealth and telemedicine and its coverage laws independently, and no 2 states are alike in their definitions and regulations [11]. While some states alternate in their use of terms such as telehealth and telemedicine, other states use them interchangeably and even add a variety of terms with a tele- prefix to refer to a remotely delivered version of a term [11].

We found it instructive to compare definitions across US states when examining how significant the framing of telehealth vernacular can be. The term telehealth may be effective in Missouri, defined in a way that encompasses a variety of visit types, but because Maryland might have a different definition for the term, it may not be as effective there. For example, the State Telehealth Laws and Reimbursement Policies Report 2021 shows only 15 states cover audio-only visits, whereas all 50 states reimburse for live video visits [11].

These nonstandard definitions and regulations lead to difficulty in designing and implementing telehealth studies across states. An effectiveness study on telehealth in Missouri, for example, would not be easily compared to a separate study done in Maryland. This discrepancy also creates confusion among patients and providers and makes policies that are needed across states or on a national level challenging to implement. Cross-state billing, for example, is not approved in many cases and poses a barrier for many clinicians who practice outside of a distant patient’s state health care network [3].

## Evaluations

Researchers across different disciplines interfacing with telehealth in their work could benefit from standardization and precision in the associated definitions [25,26]. We propose that a consensus in definitions could allow investigators to better communicate both in proposals and sharing the products of their work related to telehealth. Because telehealth comprises a large variety of interventions, it is difficult to generalize its effectiveness as different interventions can yield varied results [25]. A precise nomenclature can help researchers identify intended interventions and independent variables, leading to clearer and more precise conclusions [16-18,27,28].

Current studies underway on new telehealth programs vary in scope and outcome measures, ranging from measuring use data, such as adherence measures that count the number of app downloads, to data on the number of appointments scheduled by patients and providers, and the number of completed telemedicine visits [29]. We note that these quantitative analyses might not include qualitative perspectives that could correlate to clinical outcomes. Namely, there has been a lack of evaluations comparing the cost-effectiveness of telehealth to usual care and examining the patient experience [29-31]. Once standardized telehealth definitions are established, there will be an opportunity to better compare and understand the outcomes of in-person care compared to telehealth since the beginning of the COVID-19 pandemic. Standardized definitions have been found to be useful in other clinical research disciplines, such as for cardiovascular outcomes (eg, for metabolic syndrome and primary cardiovascular disease prevention), where studies with differing scopes can be analyzed using agreed-on basic characteristics and shared definitions [32].

The 2017 National Quality Forum report Creating a Framework to Support Measure Development for Telehealth proposed a more quality of care–driven measurement framework made to guide outcome measures about overall experience and care delivery outcomes [33]. This quality of care–motivated framework includes 4 key domains: access to care, financial cost and impact, experience, and effectiveness. Subdomains were also proposed, which include a measurement framework to correlate with clinical and quality of care outcomes. The adoption of this framework would support our aim of standardizing investigations and linking them to care delivery. These domains have a direct impact on quality of care, but this framework has yet to be used as a standard practice [29].

Telehealth is evolving and expanding rapidly with new research, facets, and associated technology. Telehealth today is not the same as it was last year, especially after its expansion in the era of COVID-19. For example, with the rise of smartphone technology, mHealth today is not the same as mHealth 10 years ago [15]. For studies of telehealth to have a fair comparison, normalized definitions and classifications of the different types of care provided via telehealth are required because this can make comparisons across studies easier, both geographically and through time [29].

A large number of organizations and institutions are defining telehealth and doing so on terms that suit their stakeholders, which has created a unique challenge. It is much easier to change a single normative definition than several definitions all at once. Now that organizations such as the US Centers for Disease Control and Prevention, the Office of the National Coordinator for Health Information Technology, the American Academy of Family Physicians, the American Medical Association, state and federal payors, as well as local private payors and institutions are all developing their own definitions, this exacerbates the complexity of developing unified definitions [9,11,13,34,35]. We risk definitions not being updated on the same cycle and further diverging over time.

Standardized definitions will help further to evaluate the system and measure and follow impacts on equity. There are observed trends in the adoption of telehealth from a patient perspective [4,23,36-38]. As Rodriguez shows, there are differences in the use of telehealth services across various ethnic groups [23]. The motivation or explanation from a patient stakeholder perspective warrants further investigation to guide equitable approaches. Failure to account for the patient perspective, especially in health disparity populations and underrepresented populations, can lead to further marginalization and exacerbation of health inequalities [39]. On an enterprise level, institutional resources can vary, which can directly impact the resources available to clinicians and providers and patterns of technology adoption.

There are many different facets of inequity, including structural racism, wealth, sexism, geographic location, and more, which contribute to overall health inequity and social determinants of health [40-42]. We propose that clear definitions of available telehealth services, though they may not solve all forms of inequity, have the potential to reduce misunderstandings, miscommunications, and confusion, all of which contribute to a lack of access to telehealth services. More attention is needed to understand the impact that interventions addressing patient preferences regarding digital health, digital literacy, and technological access will have on equity.

As telehealth rapidly evolves, we propose that attention should be paid to maintaining equity in part through clear definitions and acknowledging the access differentials that may vary across institutions and populations.

## Conclusions

Consensus is critical in terms of both equitable access and clinical and other research on the growing field of telemedicine and telehealth. Our scope of interest is in these areas, recognizing the importance of definitions across the institutional level, including interactions with third-party payors, which we have not addressed in this work. Looking to the future, health care organizations can consider consistent and agreed-on standardized definitions to benefit all stakeholders by providing clearer communications, comparable policies, precise and controlled evaluations, and by supporting the delivery of equitable care. This consensus is especially important now as telehealth could be taking on a larger role as part of routine clinical access to care. It is important that we agree on a common nomenclature soon to make full use of its benefits and prevent future backtracking.

## Abbreviations

WHO: World Health Organization
